# Effect of a diet containing folate and hazelnut oil capsule on the methylation level of the *ADRB3* gene, lipid profile and oxidative stress in overweight or obese women

**DOI:** 10.1186/s13148-017-0407-6

**Published:** 2017-10-13

**Authors:** Raquel Patrícia Ataíde Lima, Rayner Anderson Ferreira do Nascimento, Rafaella Cristhine Pordeus Luna, Darlene Camati Persuhn, Alexandre Sérgio da Silva, Maria da Conceição Rodrigues Gonçalves, Alessio Tony Cavalcanti de Almeida, Ronei Marcos de Moraes, Eliseu Verly Junior, Emmanuelle Fouilloux-Meugnier, Hubert Vidal, Luciano Pirola, Marciane Magnani, Naila Francis Paulo de Oliveira, Patrícia Oliveira Prada, Maria José de Carvalho Costa

**Affiliations:** 1 Graduate Program in Nutritional Sciences, Health Sciences Center (Centro de Ciências da Saúd-CCS), Federal University of Paraíba (Universidade Federal da Paraíba), João Pessoa, Brazil; 2 Graduate Program in Molecular and Human Biology, Center of Natural and Exact Sciences (Centro de Ciências Exatas da Natureza-CCEN), Federal University of Paraíba, João Pessoa, Brazil; 3 Graduate Program in Nutritional Sciences, CCS, Federal University of Paraíba, João Pessoa, Brazil; 4 Graduate Program in Cellular and Molecular Biology CCEN, Federal University of Paraíba, João Pessoa, Brazil; 5 Department of Economics, Center for Applied Social Sciences (Centro de Ciências Sociais Aplicada-CCSA), Federal University of Paraíba, João Pessoa, Brazil; 6 Graduate Program in Decision Models in Health, CCEN, Federal University of Paraíba, João Pessoa, Brazil; 7 Department of Epidemiology, Institute of Social Medicine, State University of Rio de Janeiro (Universidade Estadual do Rio de Janeiro), João Pessoa, Brazil; 8 University of Lyon 1, CARMEN-Vileurbanne Laboratory, Villeurbanne, France; 9 Graduate Program in Nutritional and Sport Sciences and Metabolism (Ciências da Nutrição, do Esporte e Metabolismo-CNEM), School of Applied Sciences (Faculdade de Ciências Aplicadas-FCA), State University of Campinas (Universidade Estadual de Campinas–UNICAMP), Campinas, Brazil; 10 Present Address: Pós-graduação em Ciências da Nutrição, Centro de Ciências da Saúde, NIESN–Núcleo Interdisciplinar de Estudos em Saúde e Nutrição/Universidade Federal da Paraíba, Castelo Branco, João Pessoa, PB 58059-900 Brazil

**Keywords:** *ADRB3*, Obesity, DNA methylation, Biochemical analyses, Diet

## Abstract

**Background:**

Studies of genes that play an important role in the development of obesity are needed, especially studies focusing on genes that regulate food intake and affect nutrient metabolism. For example, the beta-3 adrenergic receptor (*ADRB3*) responds to noradrenaline and mediates lipolysis in adipocytes.

**Methods:**

This was a controlled intervention study involving 40 overweight and obese adult women in which food intake, anthropometric measurements, biochemical analyses, and methylation levels of the *ADRB3* gene were evaluated before and after intervention. The individuals were randomized into four groups: group 1 (G1) received 300 g of vegetables and legumes containing on average 191 μg/day of folate and 1 hazelnut oil capsule; group 2 (G2) received 300 g of vegetables and legumes containing on average 191 μg/day of folate and 1 placebo capsule; group 3 (G3) received 300 g of vegetables and legumes containing on average 90 μg/day of folate and 1 hazelnut oil capsule; and individuals in group 4 (G4) were only followed-up and maintained their regular dietary habits. Statistical analysis was performed using analysis of variance (ANOVA), Student’s *t* test and simple regression, using *STATA 13* software.

**Results:**

In the total sample, after the intervention, the women classified as overweight and obese did not present weight loss, and there was a reduction in the methylation levels of the *ADRB3* gene and malondialdehyde, as well as an increase in high-density lipoprotein cholesterol and total antioxidant capacity.

**Conclusions:**

The beneficial effect of the intake of a hazelnut capsule on the methylation levels of the *ADRB3* gene was demonstrated for the first time.

**Trial registration:**

ClinicalTrials.gov, NCT 02846025

## Background

Obesity is a metabolic disease that is rapidly increasing around the world. Recent World Health Organization data show that over one third of adults over 18 years of age (38% of men and 40% of women) are overweight, with women in all regions being more prone to obesity than men [1].

In recent decades, obesity has been considered a result of an imbalance between energy intake and expenditure that is driven by easy access to high-calorie foods and reduced energy expenditure [2].

Other factors related to the prevalence of obesity are sleep disorders, endocrine disorders, oxidative stress, inflammation, increased maternal age, microbiota, and genetics [3].

In addition to genetic factors, epigenetic mechanisms contribute to the unexplained inheritance of obesity and fat distribution [4]. These epigenetic changes have been associated with the effects of diet composition on health and disease or the long-term effects of gene-environment interactions [5, 6]. Folate is a nutrient known to participate in DNA methylation and nucleotide reactions of biosynthesis and is also involved in the formation of methyl groups, which serves as a methyl donor in DNA methylation. Another component that possibly modulates the epigenetic profile is fat and its fractions [4].

There are many important genes involved in the development of obesity whose expression is under the influence of epigenetic regulation. Recent studies have focused on evaluating changes in the methylation pattern of specific obesity-related genes.

DNA methylation represents one of the most important epigenetic mechanisms for regulating gene expression and is the most widely studied thus far. This mechanism involves the covalent attachment of a methyl group to the 5′ position of the cytosine present in the genome of the DNA sequence by DNA methyltransferases (DNMTs) [1]. This methylation is the only known modification that targets DNA itself and is generally associated with gene silencing [7].

There is increasing evidence that gene methylation may contribute to obesity. In fact, studies of the methylation of candidate genes in animal and human models have demonstrated methylation changes in promoters of several genes that are involved in obesity, appetite control, and/or metabolism [8], namely, the beta-3 adrenergic receptor (*ADRB3*), which responds to noradrenaline and mediates lipolysis in adipocytes [9].

In this context, the present study aims to evaluate the effect of a diet containing folate and hazelnut oil capsule on the methylation levels of the *ADRB3* gene*,* lipid profile and oxidative stress in overweight and obese women.

## Methods

### Study design

This is a double-blind, placebo-controlled, intervention study linked to a population-based study titled “II Ciclo de Diagnóstico e Intervenção da Situação Alimentar, Nutricional e das Doenças não Transmissíveis mais Prevalentes da População do Município de João Pessoa/PB (II DISANDNT/JP) [Cycle II of Diagnosis and Intervention of the Food, Nutritional and Non-Communicable Diseases Statuses of the Population of the Municipality of João Pessoa/PB (II DISANDNT/JP)”, which was conducted between May 2015 and May 2016. Participants were selected from the sample of overweight and obese adults with the following inclusion criteria.

The following women were included: adult women aged 20 to 59 years, who were overweight or obese (body mass index (BMI) 25.0–35.0 kg/m^2^), with different socioeconomic levels, and who were users or non-users of medications and had a preserved cognitive status. The exclusion criteria included alcoholism, smoking, neuropsychiatric disorders, use of drugs known to interfere with folic acid metabolism (in the last 3 months), use of multivitamin or mineral supplements, use of anorexigenic substances or of anabolic substances, chronic diseases affecting the endocrine and metabolic system, pregnancy, plans to become pregnant, and loss of weight during the study period.

After screening, 40 adult female subjects were selected by convenience sampling and were properly instructed regarding the study objectives according to ethical guidelines; the subjects consented to participate by signing an informed consent form. The study was approved by the Research Ethics Committee of the CCS/UFPB, under the protocol number 0569/15 and registered in clinical trials under NCT 02846025.

### Experimental protocol

After the eligibility criteria were assessed, the women were instructed to maintain the same weight, eating habits, and levels of physical activity that were found during the baseline evaluation [10] and also received an individual diet plan 1 week before starting the dietary intervention (plateau week). All of the individuals’ medical treatments remained unchanged throughout the study. At the end of the study, all participants received nutritional counseling for weight loss.

To calculate energy expenditure, the Dietary Reference Intake (DRI) formulas for maintaining body weight were used. Macronutrients were distributed according to the recommendations of the American Heart Association (AHA) [11], and the calculation and analysis of the nutrients present in the recommended diet were performed using the food equivalent system proposed by Costa [12]. The diet contained the following: carbohydrates: 45–65% (recommended level of 55%), protein: 10–35% (recommended level of 15%), and total fat: 25–35% (recommended level of 30%).

In the second week of the study, participants were randomized into four groups: group 1 (G1), in which subjects received 300 g of vegetables and legumes containing on average 191 μg/day of folate and 1 hazelnut oil capsule (25 g); group 2 (G2), in which the subjects received 300 g of vegetables and legumes containing, on average, 191 μg/day of folate and 1 placebo capsule; group 3 (G3), in which subjects received 300 g of vegetables and legumes containing on average 90 μg/day of folate and 1 hazelnut oil capsule (25 g); and group 4 (G4), in which subjects were only followed-up and maintained their regular dietary habits. To reach the folate concentrations planned for each group, the following foods were used: lentils, soybeans, corn, peas, carrots, zucchini, lettuce, chard, beetroot, broccoli, cauliflower, tomato, and cucumber.

The hazelnut oil capsule that was offered was composed basically of monounsaturated fat (68%), rich in oleic acid.

Each group consisted of 10 women, who received daily vegetables and legumes containing folate for a total period of 8 weeks.

### Nutritional and food assessment

Weight and height were measured in triplicate, and the average of the three values was used. The BMI was then calculated as the body weight (kg) divided by the squared body height (meters), and the cut-off points recommended by the World Health Organization (WHO) were used [13]. Waist circumference (WC) was used to determine abdominal obesity, with the AHA cut-off point of ≥ 88 cm [14].

To evaluate the regular food intake of the individuals, two 24-h dietary recalls (24HR) were performed, with a 15-day interval from the beginning of the intervention. The 24HR aimed to determine the dietary habits of the individuals and thus define the menu that would be implemented and to compare the intake at the end of the study. After the plateau week, a third 24HR was performed to check adherence to the nutritional counseling.

At the end of the 8 weeks of intervention, a fourth 24HR was performed to analyze the regular intake of calories and nutrients to determine whether the subjects adhered to the recommendations for the change in food intake in general and in folate intake after intervention.

To complete the 24HR, a food photograph album with household measures was used and was based on the actual weight of the average food intake validated for this population, thus minimizing possible biases of this method [15, 16].

The foods were analyzed by the nutrition software Dietwin, and the multiple source method (MSM) was used to estimate the regular intake of the individual from repeated measurements in a determined period; the variation in intake was not affected by the method [17].

### Biochemical analysis

The biochemical analyses were performed twice: during the selection of the participants and after the end of the nutritional intervention. In both analyses, the levels of total cholesterol (TC) and its fractions (low-density lipoprotein (LDL) and high-density lipoprotein (HDL)), triglycerides, malondialdehyde (MDA), total antioxidant capacity (TAC), homocysteine, and vitamin B12 were measured. The blood samples were collected after a 12-h fast at home, using sterile vacuum tubes with and without anticoagulant, according to the guidelines for the use of sharp materials.

Lipid profile concentrations were determined using the turbidimetry method using a Labmax 240 premium-Labtest automated biochemical analyzer. For analysis of antioxidant activity through the plasma MDA and serum TAC, we used a previously described protocol [18]. Homocysteine levels were quantified using the high-performance liquid chromatography (HPLC) method [19]. The serum concentrations of folic acid and vitamin B12 were measured by chemiluminescence and an electrochemiluminescence immunoassay, respectively.

### Methylation levels

DNA methylation in genomic DNA from blood was quantified at the CarMeN/Université de Lyon1-France Laboratory.

The blood was chosen for the analysis as it is a metabolically active tissue, with an important role in the adverse inflammatory and vascular consequences of adiposity, and is widely used for clinical diagnostic purposes [20].

Whole blood was obtained before and after the intervention. The blood samples were obtained at 08:30 am ± 10 (min) in all patients and in both conditions (before and after intervention) to avoid a potential sampling time effect. DNA was isolated with a QIAamp DNA Mini kit (Qiagen, Valencia, CA, USA), and the DNA concentrations of the samples were determined using a Qubit® dsDNA HS Assay Kit. Genomic DNA was modified by bisulfite and amplified by AmpliTaq Gold® DNA Polymerase (Applied Biosystems, California 94404, USA) using the sequence described in Table 1. The PCR program consisted of an initial enzymatic activation at 95 °C for 10 min, followed by 50 cycles of 45 s at 95 °C, 45 s at 60 °C and 45 s at 72 °C and a final extension at 72 °C for 10 min.

**Table 1:** Primers used to analyze the methylation status

Gene	Primers		Annealing temperature
*ADRB3*	F	5′CCTTCCTTCTTTCCCTACCG3′	64 °C
	R	5′TGGTCTGGAGTCTCGGAGTC3′	

*F* forward primer, *R* reverse primer

The analysis of the results was performed with PyroMark Q24 software (Qiagen, Hilden, Germany).

### Statistical analysis

For proposed objectives, a descriptive analysis of the characteristics of the sample was performed first using the mean and standard deviation before and after intervention. Data were assessed for normality using the Lilliefors test, a modification of the Kolmogorov-Smirnov test [21]. Statistical analysis was performed with the STATA 13 software. To analyze the initial and final values after dietary intervention, the normal variables were analyzed using Student’s *t* test. Moreover, analysis of variance (ANOVA) was used to compare the effects of the intervention between the four groups, and the relationship of methylation levels per group was evaluated by simple linear regression.

All analyses were performed with log10-transformed methylation percentages to obtain normality. For all statistical analyses, values of *p* < 0.05 were considered significant.

## Results

The study included 40 women classified as overweight or obese. Means and standard deviations are presented in Table 2; the anthropometric measurements, DNA promoter methylation, and biochemical parameters were evaluated before and after the dietary intervention.

**Table 2:** Anthropometric characteristics, methylation level, lipid profile and oxidative stress of women before and after intervention

Parameter	Before intervention		After intervention		
	Mean	SD	Mean	SD	*p* value
Weight (kg)	77.7	14.1	74.4	14	0.4013
Height (m)	1.59	1.1	1.59	1.1	–
BMI (kg/m^2^)	30.5	5.3	29.2	5.1	0.3792
WC (cm)	0.94	0.12	0.90	0.12	0.3015
HC (cm)	1.14	0.12	1.01	0.11	0.2288
Waist-to-height ratio (WHtR; cm/m)	0.59	0.8	0.57	0.7	0.3471
Methylation level (%)	42.2	18.1	29.1	14.1	0.0006*
Total cholesterol (mg/dl)	201.6	46.2	194.8	48.1	0.5818
HDL-C (mg/dl)	44.3	9.6	50.7	9.2	0.0118*
LDL-C (mg/dl)	122.7	43.7	119.9	40.1	0.8103
Triglycerides (mg/dl)	146.7	80.6	150.5	67.7	0.8462
MDA	3.2	0.9	4	0.8	0.0029*
TAC (%)	41	13	53	12	0.0005*

It was observed, after intervention in the total sample, lower methylation levels of the *ADRB3* gene and increase in HDL-C, MDA, and TAC values.

Table 3 shows the methylation levels and food intake distributed by intervention group, with values for the following nutrients: folate, total fat, saturated fat, monounsaturated fat, polyunsaturated fat, and trans fat. Regarding weight, the groups that had lower levels of methylation had lower weights.

**Table 3:** Methylation levels of the *ADRB3* gene, anthropometric data, and regular food intake of women of the post-intervention groups

Variables	Group 1 Mean ± SD	Group 2 Mean ± SD	Group 3 Mean ± SD	Group 4 Mean ± SD	*p* value
Methylation levels (%)	25.3 ± 5.2	34.7 ± 3.5	18.1 ± 2.6	38.3 ± 3.5	0.0030*
Weight	70.15 ± 2.93	80.56 ± 6;60	73.48 ± 2.08	75.91 ± 2.62	0.3207
Calories	1444.1 ± 114.5	1512.8 ± 67.7	1454.8 ± 100.1	1637.2 ± 100.6	0.4863
Folate	311.2 ± 7.1	335.4 ± 9.5	170.1 ± 14.0	96.4 ± 2.05	0.000*
Total fat (g)	48.9 ± 3.57	42.9 ± 2.61	49.57 ± 4.36	43.64 ± 4.1	0.4667
Total fat (%)	31.06 ± 1.84	25.5 ± 1.17	31.4 ± 2.92	24.2 ± 1.89	0.0037*
Saturated fat (g)	21.48 ± 2.22	21.32 ± 2.0	26.6 ± 1.91	19.24 ± 1.7	0.0748
Saturated fat (%)	13.8 ± 1.56	12.6 ± 1.08	16.9 ± 1.41	11.3 ± 1.58	0.0551
Monounsaturated fat (g)	33.6 ± 1.46	14.2 ± 0.41	36.02 ± 1.61	26.41 ± 2.98	0.0000*
Monounsaturated fat (%)	21.6 ± 1.26	15.3 ± 2.20	23.1 ± 1.51	8.6 ± 0.45	0.0000*
Polyunsaturated fat (g)	12.81 ± 1.7	6.76 ± 0.58	19.51 ± 1.66	9.21 ± 0.95	0.0000*
Polyunsaturated fat (%)	8.14 ± 0.96	5.3 ± 0.74	12.4 ± 1.34	4.05 ± 0.31	0.0000*
Trans fat (g)	0.58 ± 0.1	0.54 ± 0.08	0.55 ± 0.08	0.53 ± 0.11	0.9859

For the calorie intake, no difference was obtained even with dietary intervention, and individuals did not consume the recommended values of folate (400 μg/day, according to the DRI). For total fat, the groups were within the recommended limit (25–30% fat corresponding to energy consumed); for saturated fat, all subjects consumed levels above the recommended (the guidelines recommend a saturated fat intake < 10%); for monounsaturated fat, the groups consumed an adequate amount (> 15%), with the exception of group 4; for polyunsaturated fat, groups 2 and 4 did not reach the recommended amount (> 10%).

Group 3 showed a decrease in methylation levels after the intervention (Table 4). Regarding weight, hip circumference and BMI, no significant differences were obtained between the groups (data not shown). However, WC was 1.2 cm lower in group 3 than in the other groups, and waist-to-height ratio (WHtR) decreased by 7.92 cm/m.

**Table 4:** Simple regression analysis of methylation levels, anthropometric measurements, lipid profile, and oxidative stress for the different intervention groups

Simple regression				
Methylation levels				
	Coefficient	95% CI	*t* statistics	*p* value
Group 1	− 0.13	− 0.24 ± − 0.02	− 2.37	0.023*
Group 2	− 0.03	− 0.14 ± 0.07	− 0.66	0.514
Group 3	− 0.20	− 0.31 ± − 0.09	− 3.69	0.001*
Waist				
	Coefficient	95% CI	*t* statistics	*p* value
Group 1	− 3.8	− 13.32 ± 5.72	− 0.81	0.424
Group 2	− 1.6	− 11.12 ± 7.92	− 0.34	0.735
Group 3	− 1.2	− 21.52 ± − 2.47	− 2.55	0.015*
WHtR				
	Coefficient	95% CI	*t* statistics	*p* value
Group 1	− 1.77	− 8.35 ± 4.80	− 0.55	0.580
Group 2	− 0.65	− 7.23 ± 5.92	− 0.20	0.841
Group 3	− 7.92	− 14.50 ± − 1.35	− 2.44	0.020*
LDL				
	Coefficient	95% CI	*t* statistics	*p* value
Group 1	− 54.34	− 90.78 ± − 17.9	− 3.02	0.005*
Group 2	− 50.32	− 86.76 ± − 13.87	− 2.80	0.008*
Group 3	− 54.78	− 91.22 ± − 18.33	− 3.05	0.004*
HDL				
	Coefficient	95% CI	*t* statistics	*p* value
Group 1	16.6	9.24 ± 23.95	4.58	0.000 *
Group 2	6.5	− 0.85 ± 13.85	1.79	0.081
Group 3	6.9	− 0.45 ± 14.25	1.90	0.065
TAC				
	Coefficient	95% CI	*t* statistics	*p* value
Group 1	0.07	− 0.36 ± 0.17	1.32	0.195
Group 2	0.13	0.02 ± 0.23	2.48	0.018*
Group 3	0.03	− 0.07 ± 0.13	0.56	0.577
MDA				
	Coefficient	95% CI	*t* statistics	*p* value
Group 1	0.75	0.06 ± 1.43	2.21	0.034*
Group 2	0.43	− 0.27 ± 1.13	1.23	0.228
Group 3	0.31	− 0.38 ± 0.99	0.91	0.367

Regarding the lipid profile, the values of cholesterol and triglycerides did not show significant differences between the intervention groups (data not shown). However, for the LDL-C values, a decrease of 54.34, 50.32, and 54.78 (mg/dL) was observed in groups 1, 2, and 3, respectively. For oxidative stress, group 2 presented an increase in TAC (*p* = 0.018), and MDA increased in group 1 (*p* = 0.034).

Table 5 shows that the folate intake was highest in group 1. For fat intake, group 1 had the greatest decrease in total fat intake, and group 3 had the highest intake of saturated, monounsaturated, and polyunsaturated fats.

**Table 5:** Simple regression analysis of the regular intake of folate, total fat, and fat fractions for the different intervention groups

Simple regression				
Folate				
	Coefficient	95% CI	*t* statistics	*p* value
Group 1	195.7	158.4 ± 233.1	10.62	0.000*
Group 2	181.6	143.4 ± 219.8	9.63	0.000*
Group 3	107.5	69.29 ± 145.7	5.7	0.000*
Total Fat				
	Coefficient	95% CI	*t* statistics	*p* value
Group 1	− 4.38	− 9.87 ± 1.10	− 1.62	0.114
Group 2	− 6.85	− 12.46 ± − 1.23	− 2.47	0.018*
Group 3	− 7.84	− 13.45 ± − 2.22	− 2.83	0.008*
Saturated fat				
	Coefficient	95% CI	*t* statistics	*p* value
Group 1	2.24	− 3.47 ± 7.95	0.8	0.432
Group 2	2.08	− 3.62 ± 7.79	0.74	0.463
Group 3	7.4	1.68 ± 13.11	2.63	0.013*
Monounsaturated fat				
	Coefficient	95% CI	*t* statistics	*p* value
Group 1	7.19	1.85 ± 12.52	2.73	0.010*
Group 2	− 12.21	− 17.54 ± − 6.87	− 4.64	0.000*
Group 3	9.61	4.27 ± 14.94	3.65	0.001*
Polyunsaturated fat				
	Coefficient	95% CI	*t* statistics	*p* value
Group 1	3.6	1.85 ± 12.52	1.93	0.061
Group 2	− 2.46	− 17.54 ± − 6.87	− 1.32	0.195
Group 3	10.3	4.27 ± 14.94	5.54	0.000*

Comparing the results by group before and after intervention showed that there was a significant statistical difference in the methylation levels between groups 2 and 3 and in TAC between groups 1 and 3 (Table 6).

**Table 6:** Analysis of methylation levels, waist, WHtR, lipid profile, and oxidative stress, by group before and after intervention

Variables	Group 1		Group 2		Group 3		Group 4	
	Before	After	Before	After	Before	After	Before	After
Methylation levels (%)	41	25	55^a^	34^a^	35^b^	18^b^	42	38
Waist	0.95	0.92	0.97	0.95	0.9	0.84	0.96	0.96
WHtR	0.60	0.59	0.61	0.60	0.55	0.52	0.60	0.60
LDL-C	101.7	109.66	125.4	113.7	114.5	109.22	169	164
HDL-C	49.6	57.8	43	47.7	40.6	48.1	38.9	41.2
TAC (%)	35^c^	53^c^	41^d^	59^d^	46	49	40	46
MDA	3.45	4.2	3.14	3.87	3.03	3.76	3.23	3.45

^a^Significant difference between before and after intervention, *p* = 0.003

^b^Significant difference between before and after intervention, *p* = 0.007

^c^Significant difference between before and after intervention, *p* = 0.000

^d^Significant difference between before and after intervention, *p* = 0.001

Table 7 shows the results of the test that was performed to compare the post-intervention groups, and it was observed that when groups 2 and 3 were obtained, differences in methylation levels (*p* = 0.0016) were observed, or in other words, group 2 had a higher mean of methylation.

**Table 7:** Comparison of post-intervention groups

	Group 1 × 2	Group 1 × 3	Group 2 × 3
	*p* value		
Methylation levels (%)	0.1558	0.2361	0.0016*
Waist	0.1671	0.3672	0.3205
WHtR	0.7752	0.0581	0.0551
LDL-C	0.7989	0.9826	0.8427
HDL-C	0.0187*	0.0158*	0.9217
TAC	0.1940	0.4943	0.1436
MDA	0.4283	0.2982	0.7242

*p < 0.005

As for the other variables, we observed differences between the means of HDL-C values, in groups 1 and 2 (*p* value = 0.0187) and in groups 1 and 3 (*p* value = 0.0158), that is, group 1 presented a higher level of HDL, post-intervention.

## Discussion

In the present study, the dietary intervention with various quantities of folate from vegetables that were close to (but lower than) the DRI, as well as with adequate amounts of monounsaturated fatty acids from the hazelnut oil capsule, did not cause a statistically significant weight loss in the total sample of overweight and obese women; the dietary intervention followed the guidelines for weight maintenance. The results also showed a reduction in the methylation levels of the *ADRB3* gene and an increase in the HDL-C values, as well as a decrease in the MDA and an increase in TAC, with both of the latter values being used to evaluate oxidative stress.

The most relevant result was found in group 3 (folate: 90 μg/day + hazelnut oil capsule) after the intervention, when the methylation levels decreased (18.1%). According to Jacobsen [22], values below 25% are considered low levels of methylation (Table 1).

For weight, the women in groups 1 and 3 had lower weight and lower methylation levels. The proteins encoded by *ADRB3* genes belong to the family of beta-adrenergic receptors, which mediate the catecholamine-induced activation of adenylate cyclase through the action of G proteins. These receptors are located in adipose tissue and are involved in energy homeostasis through the mediation of lipolysis and thermogenesis rate. Thus, the genes encoding these receptors are interesting candidates to partially explain the genetic predisposition for obesity in humans [23, 24].

For the dietary intake of folate, total fat and fat fractions evaluated for each intervention group, group 2 consumed a greater daily amount of folate. For fat, groups 1 and 3 consumed higher levels of monounsaturated and polyunsaturated fat, and this difference was statistically significant.

The women in group 3 also had greater decreases in WC, WHtR, and LDL-C levels. According to Singh [25], BMI, WC, and waist-to-hip ratio are considered methods to distinguish between global obesity (measured by BMI) and central adiposity (measured by WC and waist-to-hip ratio) because a study of the Chinese population demonstrated the impact of fat distribution (subcutaneous and visceral) [25], with *ADRB3* mainly being expressed in visceral adipose tissue [26].

Studies of the *ADRB3* gene are still scarce in the literature. Such studies show that this gene plays an important role in the regulating lipolysis in the brown and white adipose tissue of humans that provide free fatty acids for thermogenesis. Additionally, there is indirect evidence that *ADRB3* may participate in the regulation of body weight in humans [27], suggesting that epigenetic changes in the locus of the *ADRB3* gene may be involved in the development of obesity and its associated metabolic complications [28].

There is some evidence for epigenetic parameters related to metabolic diseases that support a relationship between genes and the environment. This type of relationship between genes and environment, epigenetics and metabolic disorders may influence the epigenome and may be one reason for the development of obesity [29, 30].

Dietary factors have been reported to affect methylation levels, and some genes such as *MC4R* and *Leptin*, have already been reported to alter the methylation patterns that result from a high-fat diet [25]. In the present study, the women with the highest intake of monounsaturated fat were those in groups 1 and 3; these women also had lower levels of methylation. In contrast, group 3 had the highest intake of polyunsaturated fat and the lowest level of methylation, suggesting that a diet rich in polyunsaturated fat would be more effective in altering the methylation patterns of genes such as *ADRB3.*

Nutrients can modify physiological and pathological processes through the expression of altered genes, and epigenetic modifications are considered a key mechanism underlying the effects of nutrition on gene expression. Polyunsaturated fatty acids, such as omega-3, work by reducing body fat and by improving pathological parameters [31]. Another beneficial effect of polyunsaturated fatty acids is their regulation of the synthesis and oxidation of fatty acids. That is, consuming the recommended levels of monounsaturated and polyunsaturated fatty acids is suggested also because doing so will lower the methylation levels of the *ADRB3* gene.

The lipid profile is associated with obesity and may be altered in individuals who are classified as overweight and obese. Guay reported that the *ADRB3* gene is associated with dyslipidemia [27]; dyslipidemia is characterized by increased blood levels of LDL-C and triglycerides and reduced levels of HDL-C [32]. Group 1 showed the highest increase in HDL-C levels, and groups 1 and 3 had the greatest decrease in LDL-C values. These values may be associated with lower levels of *ADRB3* gene methylation. The triglyceride and cholesterol values were not significantly different in the present study.

Group 2 presented the highest levels of methylation and lowest decrease in LDL-C, as well as the lowest increase in HDL-C and the lowest intake of monounsaturated and polyunsaturated fat, which are reported to improve the lipid profile and potentially prevent diet-induced dyslipidemia [32].

Biomarkers related to oxidative stress, such as MDA and TAC, have been rarely studied in terms of their association with the *ADRB3* gene in overweight and obese women. However, group 2 had the highest increase in TAC; this group had an intake (300 g) of vegetables similar to that of the other groups and greater consumption of folate (191 μg). Increased plasma TAC values are associated with higher regular intake of fruits, vegetables, and nuts [32].

## Conclusions

In conclusion, this study is the first to demonstrate the beneficial effect of the intake of hazelnut oil capsules on the methylation levels of the *ADRB3* gene; however, the group with the highest folate intake was the group with the highest levels of methylation. Group 3 had the lowest methylation levels, the largest reductions in WC, WHtR, LDL-C, and total fat intake and the highest intake of monounsaturated and polyunsaturated fats. The data presented in this study show that the methylation levels were influenced by dietary factors, such as the intake of monounsaturated and polyunsaturated fats, that contributed to the reduction in the methylation levels of the *ADRB3* gene. The results of this study may be used in the future for the prevention and management of obesity-related complications.
